# Supplementary material for: Distinct spatial coordinate of visual and vestibular heading signals in macaque FEFsem and MSTd
Source: eLife. 2017 Nov 14;6:e29809. doi: 10.7554/eLife.29809 (PMC5685470; doi:10.7554/eLife.29809)
Supplement: Transparent reporting form [file elife-29809-transrepform.docx]

***eLife’s*** **transparent reporting form**

We encourage authors to provide detailed information *within their submission* to facilitate the interpretation and replication of experiments. Authors can upload supporting documentation to indicate the use of appropriate reporting guidelines for health-related research (see [EQUATOR Network](http://www.equator-network.org/%20)), life science research (see the [BioSharing Information Resource](https://biosharing.org/)), or the [ARRIVE guidelines](http://www.plosbiology.org/article/info:doi/10.1371/journal.pbio.1000412) for reporting work involving animal research. Where applicable, authors should refer to any relevant reporting standards documents in this form.

If you have any questions, please consult our Journal Policies and/or contact us: [editorial@elifesciences.org](mailto:editorial@elifesciences.org).

**Sample-size estimation**

- You should state whether an appropriate sample size was computed when the study was being designed
- You should state the statistical method of sample size computation and any required assumptions
- If no explicit power analysis was used, you should describe how you decided what sample (replicate) size (number) to use

Please outline where this information can be found within the submission (e.g., sections or figure legends), or explain why this information doesn’t apply to your submission:

1. In electrophysiology and psychophysics, we got our results from two monkey subjects (Figure 1, E label; Figure 6, D label; Line 304; Line 512). In electrophysiology, each tuning function was obtained for at least 3 repetitions, whereas majority of the neurons (92%) were collected for larger than five repetitions (Line 589-590). In psychophysics, psychometric functions were obtained for more than 30 repetitions (Figure 6, B & C label, Line 784).

2. For DI measured under 20°eccentricity in FEFsem and MSTd, this part of experiment includes 171 neurons significantly tuned to visual optic flow and 71 neurons significantly tuned to vestibular inertial motion in FEFsem. In MSTd, 99 and 54 neurons significantly tuned to visual optic flow and vestibular inertial motion are included in the final dataset (Figure 2, B & C; Figure 3, C & D; Line 108-110).

3. For DI measured under different eye positions (10°, 20°, 30°), 38 neurons are included, and data are pooled across areas and stimuli conditions, Supplementary Figure 1B; Line 159-160).

4. There are 54 FEFsem neurons and 51 MSTd neurons tested under the smooth pursuit condition (Figure 4 C; Line 230). Compared to the eccentric fixation condition on a cell by cell basis, there are 36 FEFsem and 32 MSTd neurons under both stimuli conditions (Figure 4D label, Line 758-759).

5. For conditions without motion parallax condition, there are 38 FEFsem neurons and 34 neurons in MSTd during the varied eye-position protocol (Figure 5B label, Line 765), and 39 FEFsem and 38 MSTd neurons during the smooth pursuit protocol (Figure 5E label, Line 772). On a cell by cell basis, there are 27 FEFsem and 17 MSTd neurons tested under both with and without motion parallax during the varied eye-position protocol (Figure 5C label, Line 769-770), and 24 FEFsem and 29 MSTd neurons during the smooth pursuit protocol (Figure 5F label, Line 772).

6. After active heading estimation training, we collected 65 FEFsem and 68 MSTd neurons tested under the eccentric fixation task (fixation only, Figure 7A, label, Line 795-796). On a cell by cell basis, we measured spatial coordinate of 36 FEFsem and 35 MSTd neurons under both fixation only and active estimation tasks (Figure 7C label, Line 802).

**Replicates**

- You should report how often each experiment was performed
- You should include a definition of biological versus technical replication
- The data obtained should be provided and sufficient information should be provided to indicate the number of independent biological and/or technical replicates
- If you encountered any outliers, you should describe how these were handled
- Criteria for exclusion/inclusion of data should be clearly stated
- High-throughput sequence data should be uploaded before submission, with a private link for reviewers provided (these are available from both GEO and ArrayExpress)

Please outline where this information can be found within the submission (e.g., sections or figure legends), or explain why this information doesn’t apply to your submission:

1. Experiments were performed daily. On average, a single neuron was isolated and recorded in each day. Neurons were collected in different positions both across days to guarantee unbiased sampling. Two monkey subjects were used in the current study with both hemispheres.

2. For heading estimation tasks, the psychometric function was obtained for at least 30 repetitions for one session in each day (Figure 6, B & C label, Line 784). During the recording experiment, each stimulus was repeated for at least 3 times (Line 589), but typically more than 5 times (Line 590, 626). Trials were discarded when the monkeys broke fixation.

**Statistical reporting**

- Statistical analysis methods should be described and justified
- Raw data should be presented in figures whenever informative to do so (typically when N per group is less than 10)
- For each experiment, you should identify the statistical tests used, exact values of N, definitions of center, methods of multiple test correction, and dispersion and precision measures (e.g., mean, median, SD, SEM, confidence intervals; and, for the major substantive results, a measure of effect size (e.g., Pearson's r, Cohen's d)
- Report exact p-values wherever possible alongside the summary statistics and 95% confidence intervals. These should be reported for all key questions and not only when the p-value is less than 0.05.

Please outline where this information can be found within the submission (e.g., sections or figure legends), or explain why this information doesn’t apply to your submission:

Every statistical analysis methods and exact information were listed within the submission (Line 126, 128, 129,131-132, 134-137, 140-141, 148, 166-167, 170-172, 213-215, 221-222, 235-240, 242, 244-247, 275-276, 284-286, 293-295).

(For large datasets, or papers with a very large number of statistical tests, you may upload a single table file with tests, Ns, etc., with reference to sections in the manuscript.)

**Group allocation**

- Indicate how samples were allocated into experimental groups (in the case of clinical studies, please specify allocation to treatment method); if randomization was used, please also state if restricted randomization was applied
- Indicate if masking was used during group allocation, data collection and/or data analysis

Please outline where this information can be found within the submission (e.g., sections or figure legends), or explain why this information doesn’t apply to your submission:

**Additional data files (“source data”)**

- We encourage you to upload relevant additional data files, such as numerical data that are represented as a graph in a figure, or as a summary table
- Where provided, these should be in the most useful format, and they can be uploaded as “Source data” files linked to a main figure or table
- Include model definition files including the full list of parameters used
- Include code used for data analysis (e.g., R, MatLab)
- Avoid stating that data files are “available upon request”

Please indicate the figures or tables for which source data files have been provided:

Figure 2B, Figure 2C, Figure 4C, Figure 5B, Figure 5E, Figure 7A
